# The impact of introducing multidisciplinary care assessments on access to rheumatology care in British Columbia: an interrupted time series analysis

**DOI:** 10.1186/s12913-022-07715-x

**Published:** 2022-03-11

**Authors:** Ross Duncan, Lucy Cheng, Michael R. Law, Kam Shojania, Mary A. De Vera, Mark Harrison

**Affiliations:** 1 Faculty of Pharmaceutical Sciences, University of British Columbia, 2405-4625 Wesbrook Mall, Vancouver, BC V6T 1Z3 Canada; 2 Arthritis Research Canada, Richmond, BC Canada; 3 BC Academic Health Science Network, Vancouver, BC Canada; 4 School of Population and Public Health, Centre for Health Services and Policy Research, University of British Columbia, Vancouver, BC Canada; 5 Department of Medicine, Division of Rheumatology, University of British Columbia Faculty of Medicine, Vancouver, BC Canada; 6 Centre for Health Evaluation and Outcome Sciences, St. Paul’s Hospital, Vancouver, BC Canada

**Keywords:** Fee for service, Canada, Policy, Interrupted time series, Rheumatology

## Abstract

**Background:**

In 2011 the British Columbia (BC) Ministry of Health introduced a new fee-for-service billing code that allowed “Multidisciplinary Care Assessment” (MCA). This change has the potential to change access to and quality of care for patients. This study aimed to explore the impact on access to rheumatology services in the province.

**Methods:**

Fee-for-service rheumatology billings were evaluated for each rheumatologist 2 years before and after use of the MCA code. Numbers of 1) unique patients and 2) services provided per month were used as proxy measures of access to care. A multiple-baseline interrupted time series model assessed the impact of the MCA on levels and trends of the access outcomes.

**Results:**

Our analysis consisted of 82,360 patients cared for by 26 rheumatologists who billed for an MCA. In our primary analysis we observed a sustained increase in the mean number of unique patients of 4.9% (95% CI: 0.0% to 9.9%, *p* = 0.049) and the mean number of services of 7.1% (95% CI: 1.0% to 13.6%, (*p* = 0.021), per month provided by a rheumatologist, corresponding to the initial use of MCA.

**Conclusion:**

The introduction of the MCA code was associated with an initial increase in the measures of access, which was maintained but did not increase over time. Our study suggests that the use of Multidisciplinary Care Assessment can contribute to expanding and/or sustaining access to care for people with complex chronic conditions, like rheumatic diseases.

**Supplementary Information:**

The online version contains supplementary material available at 10.1186/s12913-022-07715-x.

## Background

Multidisciplinary care (MDC) teams are increasingly recognized as a potentially beneficial model of care for a myriad of conditions and contexts. This includes primary care, diabetes management, dermatology, cancer, chronic kidney disease, non-cancer pain management, stroke survivors, and rheumatology among others [1–8]. While many evaluations of MDC indicate encouraging results for quality of care and clinical outcomes [2–5, 8], research into MDC is complicated by the diversity of contexts and variations across intervention models [1, 9]. Rheumatology is a discipline that exemplifies this element of MDC, with Crossland et al. [8] finding that 9 out of 11 identified randomized controlled trials (RCT) reported clinical benefits to MDC, including reduced pain, improved self-management, improved emotional and psychological wellbeing, and reduction in inflammatory disease activity.

Inflammatory rheumatic disease typically involves chronic pain, inflammation and damage of joints and connective tissues, and, in severe cases, organ damage, and early intervention is associated with improved prognosis [10, 11]. Consequently, timely access to care, and ongoing management of disease is essential to slowing its progress, improving patient quality of life and to reducing long-term healthcare burden on the government payer. Unfortunately, there has been concern regarding workforce supply of rheumatologists in Canada broadly [12, 13], and in British Columbia (BC) in particular [14, 15]. A 2010 survey conducted by the BC Society of Rheumatologists (BCSR) described a “looming crisis” for BC’s rheumatology workforce; BC had approximately half the number of rheumatologists-per-capita recommended by the Canadian Rheumatology Association [14] and a high proportion of these rheumatologists were planning to retire within the next decade (24% within 5 years and another 28% within 10 years). In response, the BCSR was successful in persuading the BC Ministry of health (MoH) to fund the use MDC teams (nurse support for rheumatologists) to help bridge the gap in supply of rheumatology services [14]. The BCSR hoped that “[t]his treatment model would increase the ability of the rheumatologist to provide treatment to a wider spectrum of patients, and have an impact on the lengthy waits that exist for access to rheumatology consultation” [16].

While the benefits of MDC teams are relatively well-established, a natural experiment in shifting patterns of rheumatology care in the Canadian province of British Columbia provides the opportunity to examine a potential benefit of MDC that has not yet received much attention in the literature – the ability to support and/or expand access to care. Our hypothesis was that facilitating rheumatologist-nurse MDC in the community would increase access to services, and in the context of an apparent shortfall of supply of rheumatologists in BC, we would observe an increase in the level and trend of both these outcomes after the introduction of the new BC MoH policy. There is RCT-based evidence to suggest that nurse-led and/or nurse-supported rheumatology care is clinically equivalent to rheumatologist-only care [17, 18], is associated with improved patient satisfaction and outcomes [17–19], and can reduce costs of health care [17, 20, 21].

However, the BC policy intervention – a new fee-for-service billing code targeted at community practitioners – differs from examples of successful, institution-based nurse MDC interventions from these RCTs. The MDC interventions studied previously in RCTs include nurses with advanced training for and/or extensive experience in rheumatology [17–21], have structured protocols for intervention with a specific schedule of tasks [17, 18, 21], and had 3-monthly scheduled visits [17, 19, 20] with specialist nurses, in some cases with a nurse-led telephone helpline [19, 20]. In contrast, the MDC intervention in BC does not require the nurse to have any advanced qualifications or experience in rheumatology, could only be billed once every 6 months for each patient, and offered only broad guidance that an MDC consultation should consist of was “assessment, written treatment plan and any other counselling the patient needs for management of their particular diagnosis” [22.] It is therefore uncertain how MDC delivered through this billing code would impact care in an uncontrolled clinical context at a population-level. Whilst an uncontrolled study of three rheumatology offices in BC reported that use of the nurse-supported MDC was associated with increases in the number of patient visits provided by these clinics [16], the need for a large-scale evaluation of the impact on access remains. Therefore, we examined how the introduction of a billing code to enable nurse-supported care impacted access to rheumatology care in BC using evidence from a natural experiment at a provincial population-level with real world behavior as evidence.

## Methods

### Study context

A new billing code, now known as the Multidisciplinary Care Assessment (MCA), was approved by the BC MoH and added to the fee-for-service billing codes available to rheumatologists as G31060 in April 2011 through the Medical Services Plan (MSP). The MCA code provides an additional amount, in excess of most standard consultations, to allow a rheumatologist to pay a nurse to support care of rheumatology patients [16, 23]. In BC, the Medical Services Plan provides universal insurance to residents with no co-payment at the point of service, with fee-for-service practitioners billing the government directly.

The MCA code is restricted to rheumatology practices and is intended for patients with inflammatory diseases such as rheumatoid arthritis which require continuing management by a rheumatologist. It is not intended for the evaluation and/or management of uncomplicated rheumatologic disorders (e.g.: osteoarthritis, bursitis/tendonitis, neck and back pain). The billing code is only paid when either a Registered Nurse (RN) or Licensed Practical Nurse (LPN) is present at the consultation, and can be billed once per patient in a 6-month period, and cannot be paid in addition to other consultation codes [22]. As part of the assessment the patient should receive a written treatment plan and any counselling needed for management of their disease. The MCA code paid CAD$226 per consultation in 2019, and cannot be paid in addition to standard (code: 31,010; $209), repeat/limited (code: 31,012; $121), extended (code: G31050; $270) consultations or a subsequent office visit (code: 31,007; $87). By offering a premium over these codes, the MCA code is intended to allow rheumatologists to hire and remunerate the nurse, and is unique in Canada in allowing rheumatologists in private and community practices to provide nursing support (personal communication: K. Shojania, March 14^th^ 2021 & M. Teo, May 19^th^ 2021).

### Data sources

This study uses two population-based datasets used in BC between April 1^st^ 2009 through to March 31^st^ 2016 and held by Population Data BC [24]. Population Data BC supports the linkage of and access to individual-level, de-identified data for research. First, the Medical Services Plan (MSP) payment information file records all procedures billed via fee-for-service in BC [25]. Associated with the MSP payment information dataset, we used the BC Ministry of Health Services Central Demographics File (MSP Registration and Premium Billings, Client Roster and Census Geodata)/Consolidation file (MSP registration and premium billing) data set to describe the demographic characteristics of patients (age, sex, rurality) [26].

### Cohort definition

We used an open cohort of all people in the BC population living with rheumatic disease and receiving active care from a rheumatologist. We identified rheumatologists using two methods. First, using an anonymous pseudo-identifier provided by Population Data BC based on a list of MSP billing numbers we submitted. We obtained MSP billing numbers for all rheumatologists listed on directories of rheumatologists in BC during our study period using the directory of the College of Physicians and Surgeons of British Columbia. The second was by identifying practitioner numbers in the MSP data that billed rheumatology-specific codes (i.e. 44) in the “Physician Specialty Code” under the MSP fee schedule. Patients accessing rheumatology care were identified by services billed in MSP that are identified as rheumatology codes in the MSP fee schedule by practicing rheumatologists in BC. Patient diagnoses were based on International classification of disease (ICD) codes associated with each patient contact and classified using existing algorithms [27–30]. The algorithm evaluates the ICD-9 and 10 codes associated with patient encounters with the health system and prioritizes diagnoses from rheumatologist visits to identify a confirmed index diagnosis of rheumatic disease. Demographic characteristics of patients were based on data from the year of the indexed diagnosis date. During data cleaning, erroneous billings (identified by those set to 0 or negative service units) were removed from the MSP dataset. Prior to data cleaning, 1,210,738 rheumatology billings were identified during our study period, and 813,097 were retained after data cleaning.

Our primary analysis examined rheumatologists who “consistently” used MCA. We operationally defined “consistent” as a rheumatologist using MCA at least once per month, for every month that they billed any other rheumatology service. The latter condition was added to capture when the rheumatologist was actively practicing during months they had not used MCA, representing a discontinuity in use. Occasional months of no billing activity were taken to be periods of time-off by the rheumatologist and were not excluded but taken to be part of the secular workflow of physicians. We also conducted secondary analyses on rheumatologist behavior with respect to having “ever billed” the MCA code and “high-intensity” (using MCA at least 25 times per month, on average) using the same methods.

### Outcome measures

This study assessed the impact of MCA on two measures of access to rheumatology care: 1) the total number of unique patients (including new and existing patients) seen by rheumatologists per month, identified by one or more billing codes submitted for their care in a given month, and 2) the total number of rheumatology services (including the MCA) billed by rheumatologists: a count of all rheumatology-specific billing codes submitted by rheumatologists to MSP for referred patients. These billing codes include consultations (consultation, extended consultations for patients with chronic and complex medical needs, and repeat or limited consultations within 6 months of the last consultant visit), continuing care by a consultant, telehealth services, and two miscellaneous codes (immunosuppressant review and the MCA).

### Statistical analysis

We used a multiple-baseline interrupted time-series model on data for multiple rheumatologists, which establishes a unique baseline for each rheumatologist. The intervention occurred at different times for different rheumatologists within the different series because use of the MCA code was voluntary [31, 32]. A date of intervention (or “index date”) was defined for each rheumatologist as their first ever billing of the MCA (G31060) code, and rheumatologists with 24 months of data prior to and following this “index date” were included in the analysis, to ensure a balanced design with a sufficient data to capture secular and seasonal trends.

We used a generalized linear mixed-effects negative binomial model (GLMM) for count data with the log link and a random baseline intercept to account for heterogeneity of the study outcomes of patient and billing volumes that exists across rheumatologists. We chose a negative binomial model to correct for overdispersion [33] after assessing distributional appropriateness; the variance of the Pearson residuals from a Poisson model were more than 20 times what would be expected if the data were truly from a Poisson distribution. To account for serial correlation within rheumatologists, we used a first-order autoregressive function AR(1) in our models [34].

We computed standard errors and confidence intervals for the estimate parameters using bootstrapping, a nonparametric approach that relies on resampling with replacement (1000 samples) to approximate the distribution of parameters [35, 36]. We used the resulting bootstrap distribution to estimate the p-value and the 2.5 and 97.5 percentiles as the 95% confidence intervals for the parameters [37]. Due to differencing inherent in final GLMM specification, the results we present are the relative change, or the percentage change attributable to the policy effect compared to the counterfactual. All analyses were conducted using SAS 9.4 (SAS Institute, Cary NC).

This study was reviewed and approved by the University of British Columbia Clinical Research Ethics Board (H16-02934).

## Results

The initial cohort consisted of 152,251 patients being cared for by 77 rheumatologists between April 1^st^ 2009 through to March 31^st^ 2016. 51 (66%) rheumatologists had billed the MCA code at least once between April 2011 and March 2016. In total, the MCA code was billed 44,871 times at a cost of $9,986,749 (CAD2016) during the study period, as well as 768,226 other rheumatology billing codes costing $86,902,482 (CAD2016). Of the 51 rheumatologists who had ever billed for an MCA, 26 (51%) had two years of data available before and after their index date (when they first billed the MCA code) and could be included in the analysis. Of the remaining 25, eight did not have 24 months of data prior to their index date, six did not have 24 months of data after their index date, and 11 did not have 24 months of data available before or after their index dates. Our primary analysis consisted of 16 rheumatologists, with sufficient data before and after their index data, who consistently used the billing code at least once per month following their index date, serving 53,546 patients. Patients in our primary analysis were predominantly female (68%) with a median age of 56 years old and living in an urban area (70%); almost half (47%) had a diagnosis of inflammatory arthritis (Table 1).

**Table 1:** Characteristics of patients cared for by the 77 rheumatologists in our cohort

Total: Rheumatologists (Patients)	77 (152,251)		
Use of Multidisciplinary Care Assessment billing code:			
Yes	51 (116,447)		
No			26 (50,707)
2 year follow up pre and post first billing:			
Yes (“Ever-billed”)	26 (82,360)		
No		25 (34,087)	
Billing pattern of Multidisciplinary Care Assessment:			
1 or more per month (“Consistent”)	16 (53,546)	N/A	N/A
≥ 25 per month (“High intensity”)	14 (59,515)	N/A	N/A
Patient characteristics:			
Age (years), median (IQ)	56 (45, 68)	55 (44, 66)	57 (46, 67)
Sex:			
Female, n (%)	55,477 (68%)	24,316 (71%)	35,046 (69%)
Diagnosis:			
Inflammatory arthritis, n (%)	38,811 (47%)	20,195 (59%)	20,799 (41%)
Area of residence			
Urban, n (%)	57,503 (70%)	26,190 (77%)	36,580 (72%)
Rural, n (%)	13,509 (16%)	5,123 (15%)	4,132 (8%)
Unknown/missing, n (%)	11,177 (14%)	2,670 (8%)	9,898 (20%)

Sensitivity analyses in which the definition of intervention according to our criterion for use of the MCA code being either reduced or increased included 26 rheumatologists serving 82,360 patients (“ever-billed”) and 14 rheumatologists serving 59,515 patients (“high-intensity”), respectively.

### Unique patients per month

**Fig. 1:**
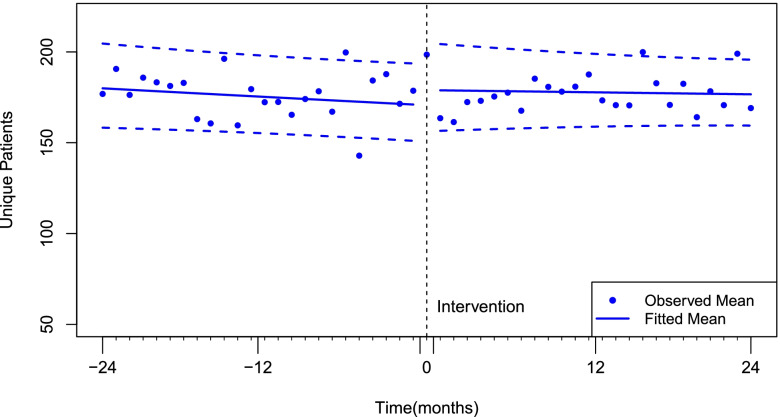
Interrupted time series model for the impact of Multidisciplinary Care Assessment billing on the number of unique patients seen by rheumatologists per month, Primary analysis (> = 1 use/month)

As shown in Fig. 1, prior to billing the Multidisciplinary Care Assessment, the mean number of patients seen per month by a rheumatologist was 171 patients (95% CI: 151, 194), and there was no statistically significant secular (pre-intervention) monthly trend (*p* = 0.141). There was a sustained increase in the mean number of unique patients per rheumatologist of 4.9% (95%CI: 0.0% to 9.9%, *p* = 0.049) at the time that rheumatologists first started billing the MCA code (Table 2). This increase represents approximately 8.5 additional patients per rheumatologist, per month (95% CI 0.3 to 17.4, *p* = 0.049) on average. However, no significant effect was found on the post-intervention trend which was estimated at an 0.2% increase per month (95% CI: -0.3% to 0.7%, *p* = 0.507).

**Table 2:** Policy effect on level and trend change in number of unique patients seen and rheumatology services provided per month, as evaluated by interrupted time series

		Change in level (β_2_)			Change in trend (β_3_)		
Outcome	Definition	Mean	95% CI	*p*	Mean	95% CI	*p*
Unique patients per month	Primary analysis:						
	1 or more per month (“Consistent”)	4.9%	(0.0%, 9.9%)	0.049	0.2%	(-0.3%, 0.7%)	0.507
	Sensitivity analysis:						
	Billed at least once (“Ever-billed”)	12.9%	(5.3%, 21.0%)	0.001	0.5%	(-0.0%, 1%)	0.052
	≥ 25 per month (“High intensity”)	8.4%	(2.7%, 14.5%)	< 0.001	0.05%	(-0.4%, 0.7%)	0.845
Services billed per month	Primary analysis:						
	1 or more per month (“Consistent”)	7.1%	(1.0%, 13.6%)	0.021	0.1%	(-0.5%, 0.7%)	0.750
	Sensitivity analysis:						
	Billed at least once (“Ever-billed”)	16.8%	(7.9%, 26.4%)	< 0.001	0.3%	(-0.2%, 0.9%)	0.239
	≥ 25 per month (“High intensity”)	8.1%	(0.2%, 15.9%)	0.044	-0.2%	(-0.8%, 0.5%)	0.485

### Number of services provided

**Fig. 2:**
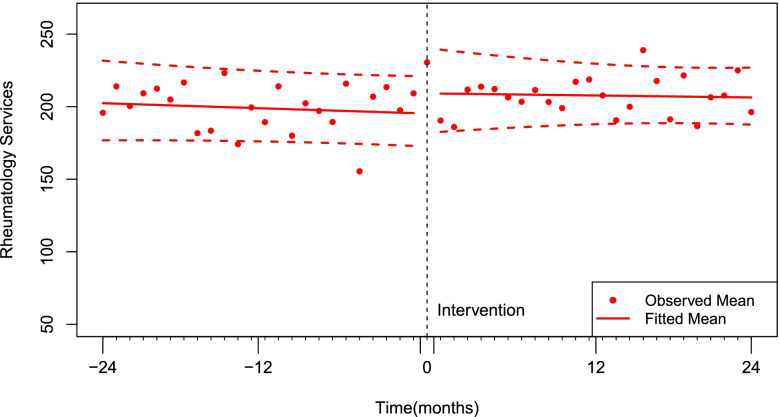
Interrupted time series model for the impact of Multidisciplinary Care Assessment billing on the number of services provided by rheumatologists per month

The mean number of services billed by a rheumatologist prior to the first time they used the Multidisciplinary Care Assessment code was 196 (95% CI 173, 221), as shown in Fig. 2. We found no statistically significant secular trend (*p* = 0.425). There was a sustained increase in the mean number of services billed per rheumatologist of 7.1% (95% CI: 1.0% to 13.6%, *p* = 0.021) corresponding with the first time a rheumatologist billed for a “MCA". This increase in billing represents an additional 14.2 services per month (95%CI: 2.0 to 27.0, *p* = 0.021) provided per rheumatologist, on average. There was no statistically significant effect on the post intervention trend of number of services provided per month, with an average increase per rheumatologist per month of 0.09% (95% CI: -0.5% to 0.7%, *p* = 0.750).

### Sensitivity analyses

Sensitivity analyses in which the definition of intervention according to use of the MCA code was relaxed (“ever-billed”) or tightened (“high-intensity”) produced consistent results, finding statistically significant changes in level at the time of intervention, albeit with larger estimates of the change. For unique patients per month, the estimated change in level was 12.9% (95% CI, 5.3% to 21.0%, *p* = 0.001) for the “ever-billed” definition and 8.4% (95% CI: 2.7% to 14.5%, *p* =  < 0.001) for the “high-intensity” definition, compared with 4.9% (95% CI 0.0% to 9.9% *p* = 0.049) in the primary analysis (“consistent”). Similarly, for services provided per month, the estimated change in level was 16.8% (95% CI: 7.9% to 26.4%, *p* =  < 0.001) for the “ever-billed” definition and 8.1% (95% CI: 0.2% to 15.9%, *p* = 0.044) for the high-intensity definition, compared with 7.1% (95% CI 1.0% to 13.6%, *p* = 0.021) in the primary analysis (“consistent”). No statistically significant impact on post-intervention trend was detected in secondary analyses for either outcome.

## Discussion

Our results show a statistically significant and sustained one-time increase in both the number of unique patients seen and rheumatology services provided by a rheumatologist, per month after the adoption of MCA. This finding was consistent in our primary and sensitivity analyses, although the magnitude of the effect varied according to the definition of use of MCA. Our analyses suggest that the impact of introducing MCA will permanently increase the number of unique patients seen per month, per rheumatologist between 4.9% (95% CI: 0.2% to 9.9%, *p* = 0.049), to 12.9% (95% CI:5.3% to 21%, *p* < 0.001) on average, depending on the definition of use of MCA by a rheumatologist. We found a similar increase in the number of services per month a rheumatologist provides after adopting MCA. The average increase in services provided ranged from 7.1% (95% CI:1.0 to 13.6%, *p* = 0.021) to 16.8% (95% CI: 7.9 to 26.4%, *p* < 0.001), depending on the definition of use of MCA by the rheumatologist.

The MCA was introduced in the context of a “looming crisis” of workforce demography [14] as a way of helping to maintain and improve access to rheumatology services for patients, which is crucial for their prognosis [14]. This is aligned with evidence from RCTs studying the efficacy of nurse-led rheumatology care in increasing access to and continuity of care [18, 38], suggesting that similar interventions like the MCA in BC, have been effective in increasing access to rheumatologists (numbers of patients seen/services provide) when introduced in practice. Further evidence from the literature suggests that satisfaction with nurse-led rheumatology care, an indicator of quality, access, and continuity of care [39], is comparable with or higher than with rheumatologist-led care [18, 19, 21, 38, 40, 41]. This increased satisfaction, linked to access, reported in the literature might be explained by more frequent contact, longer consultation times and more investigations associated with nurse support [42]. However, as the MCA introduced via a billing code in BC only pays for a nurse to see a patient every six months, which is less frequent contact than in other examples of successful nurse interventions, and is less prescriptive about the role of the nurse, it was unclear whether the efficacy seen in RCTs of nurse-led rheumatology care [21, 43–45] would be delivered in practice.

There are few studies evaluating the impact of multidisciplinary care teams, such as the MCA, on access measures directly. Two studies in BC have documented the role that nurses employed under the MCA billing code performed within a sample of three practices, and then a subsequent study of three practices compared the unique patients seen per week prior to and after the introduction of MCA in BC and patient satisfaction [16]. The first study of the MCA in BC reported that rheumatology nurses were providing patient management and counselling, training in self-injection of drugs, general education, and immunizations and tests [46], which might free up time for rheumatologists to see more patients. The subsequent study, again in 3 rheumatologist clinics in BC, reported a 74% increase in average number of patients seen per week, from 17.4 in 2009 to 30.4 in 2016 as well as patient satisfaction [16], which is consistent with the previous literature [39]. Whilst our findings suggest a smaller increase in numbers of patients seen, as well as an increase in services provided, they are consistent in suggesting that the MCA in BC has had some success in increasing access to rheumatology services for patients in BC, and that despite less frequent contact of nurses with patients, and a less prescriptive role for nurses, that some of the benefits documented in RCTs have been achieved in practice.

Any increases in access, measured by unique patients seen and services provided, should be considered in the context of the additional cost of the intervention, to understand whether the MCA led to greater efficiency in service provision. However, there are two main reasons to be cautious in using the results of this study for this purpose. Firstly, consideration of efficiency requires a stated objective. The MCA code was introduced to allow patients with specific inflammatory diseases to have additional counseling, training and education about managing their disease and therapy from rheumatology nurses alongside their regular rheumatology care [47]. Therefore, the overarching goal appears to have been to increase the quality of care to improve patient outcomes, although other potential benefits of reducing waiting times and freeing up physician time, which could translate to increased access have been acknowledged. Although access to care is increasingly identified as a key indicator of quality of care in the USA [48] and Canada [12, 49], the potential benefits of the MCA go beyond increasing access to care. Secondly, although the MCA code was billed almost 45,000 times at a cost of nearly $10 m (CAD2016) during the study period, this does not represent the true cost of the intervention; the MCA code is a substitute for other codes, offering a premium for nursing care over standard, repeat or limited, or subsequent office visits, and paying less than extended consultations. Therefore, the incremental cost of the MCA will be considerably smaller.

Whilst the addition of nurses to the care team may lead to increased costs [17, 20, 21, 38, 50, 51], there is evidence from RCTs that the use of multidisciplinary care teams, usually nurse-supported or led, appears to be clinically equivalent to rheumatologist-only care according to multiple RCTs, with equivalent or marginally better patient outcomes in multidisciplinary teams [17, 19, 21]. This is consistent with evaluations of multidisciplinary care teams within the context of primary care [52]. Furthermore, multidisciplinary rheumatologists clinics with nurses have also been reported to result in fewer contacts with other health care professionals [50]. Further research in BC should explore the impact of the MCA on patient outcomes and overall costs and cost-effectiveness to the BC Ministry of Health.

Our analysis has some limitations. While we used a population-based approach, the total population of rheumatologists in BC is small, with only 77 unique practitioners identified across the entire study period, and not all practicing continuously. Furthermore, as the use of MCA is voluntary, only a subgroup of the population of rheumatologists were included in our analysis. However, our results represent the most valid assessment of impact of the MCA on access using robust methods on the total population of rheumatologists who switched to using the MCA following the introduction of the billing code. Our analysis was also limited in several ways by the nature of the administrative data available. Secondly, there is no linkable dataset with information on the demographics of rheumatologists or regarding the context of their practice, which may affect their adoption of and use of MCA. A 2019 study on rheumatology workforce planning suggests that rheumatologists’ working hours and patient volume vary according to characteristics like their age and sex, as well as the type of practice in which they work, for example whether they run clinics as solo practitioners or work in small teams [53]. As the effect of MCA is likely to occur at the level of the entire practice, not just the rheumatologist who billed the code, evaluation would ideally occur at the clinic level. However, without this data available, it was only possible to conduct a rheumatologist-level evaluation regarding the introducing of MCA. The lack of information about the demographics and practice patterns of rheumatologists is not unique to this study, a literature review recently highlighted the need for better information on working patterns, interaction with remote communities, and the models of care used by rheumatologists [54].

Our study also has major strengths. First, the analysis was population based, meaning that all practitioners and patients providing and receiving rheumatology care under the BC health care system were available for analysis and our inclusion criteria sought to maximize the internal validity of our statistical design. Furthermore, we tested the robustness of our results to changes in the definition of intensity of use of the MCA billing code. Secondly, we used a robust, quasi-experimental statistical method, multiple-baseline segmented regression, that has been consistently used to evaluate the impact of changes in policy such as the MCA using administrative data in BC [55–57] and across the world [58–61]. By incorporating pre- and post-intervention periods, we were able to observe and adjust for secular trends, shocks, and seasonal effects that may otherwise lead to invalid results [62]. By indexing the intervention point individually for each rheumatologist, we were able to make fair comparisons of our measures of access before and after use of a nurse through the MCA using data from as many rheumatologists as possible. Short of a randomized controlled trial, the methods used in this study represent the most internally valid possible evaluation of MCA’s impact on our outcome measures.

## Conclusion

In conclusion, the introduction of multidisciplinary care by rheumatologists and nurse in BC was associated with a sustained, one-time increase in the number of unique patients seen and number of rheumatology services provided. Our study suggests that the use of Multidisciplinary Care Assessment can contribute to expanding and/or sustaining access to care for people with complex chronic conditions, like rheumatic diseases.

## Supplementary Information

**Additional file 1: Appendix A.** ITS regression plots for secondary analyses. **Appendix B.** Rheumatologist Categorization Algorithm.

## Data Availability

The data that support the findings of this study are available from Population Data BC (https://www.popdata.bc.ca/) but restrictions apply to the availability of these data, which were used for the current study under Data Stewards’ approval and Research Agreements with Data Stewards, and so are not publicly available. Data are however available from Population Data BC upon reasonable request and with permission of the Data Stewards.

## Abbreviations

AR: Autoregressive
BC: British Columbia
BCSR: British Columbia Society of Rheumatologists
CAD: Canadian Dollar
CI: Confidence Interval
ICD: International Classification of Diseases
GLMM: Generalized Linear Mixed-Effects Model
ITS: Interrupted Time Series
LPN: Licensed Practical Nurse
MCA: Multidisciplinary Care Assessment
MDC: Multidisciplinary Care
MoH: Ministry of Health
MSP: Medical Services Plan
RCT: Randomized Controlled Trial
RN: Registered Nurse
