# Supplementary material for: The impact of introducing multidisciplinary care assessments on access to rheumatology care in British Columbia: an interrupted time series analysis
Source: BMC Health Serv Res. 2022 Mar 11;22:327. doi: 10.1186/s12913-022-07715-x (PMC8915460; doi:10.1186/s12913-022-07715-x)
Supplement: Supplementary file 1 — Additional file 1: Appendix A. ITS regression plots for secondary analyses. Appendix B. Rheumatologist Categorization Algorithm. [file 12913_2022_7715_MOESM1_ESM.docx]

**Appendix A:** **ITS regression plots for secondary analyses.** Top, “Ever-Billed” Rheumatologists. Bottom, “High-Intensity” Rheumatologists.

| 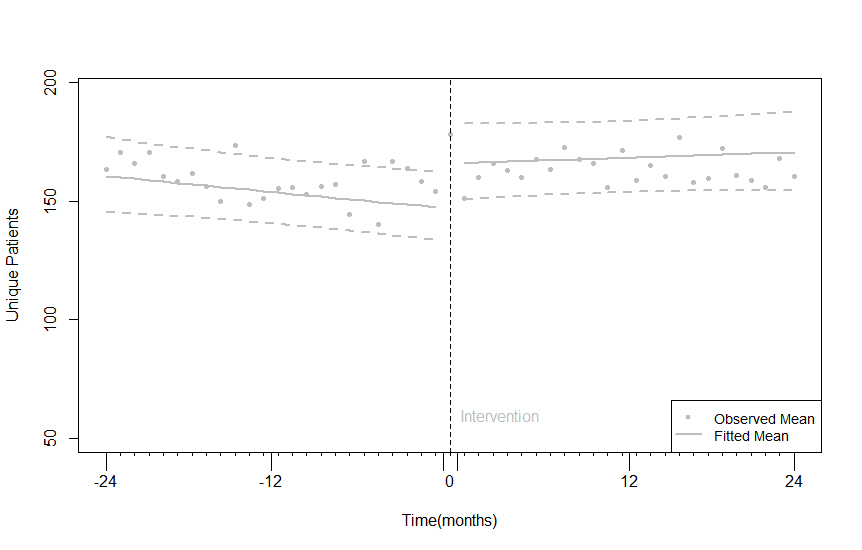 | 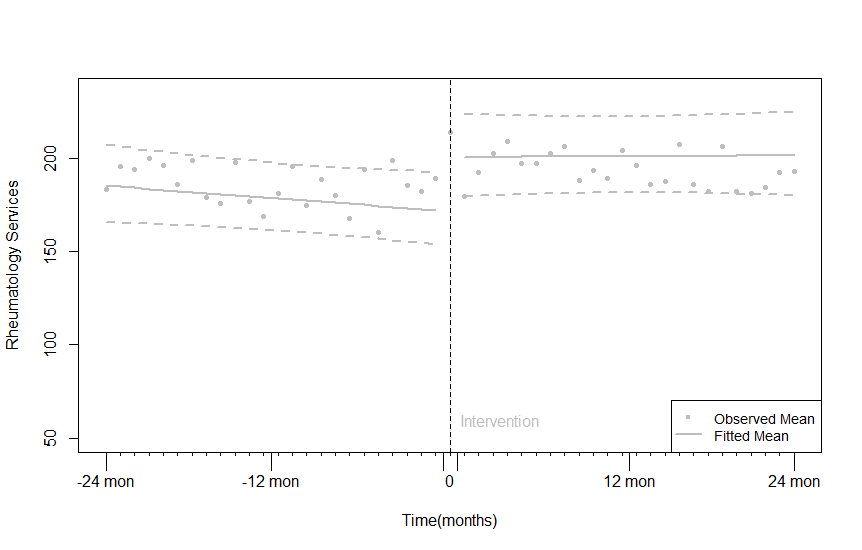 |
| --- | --- |
| 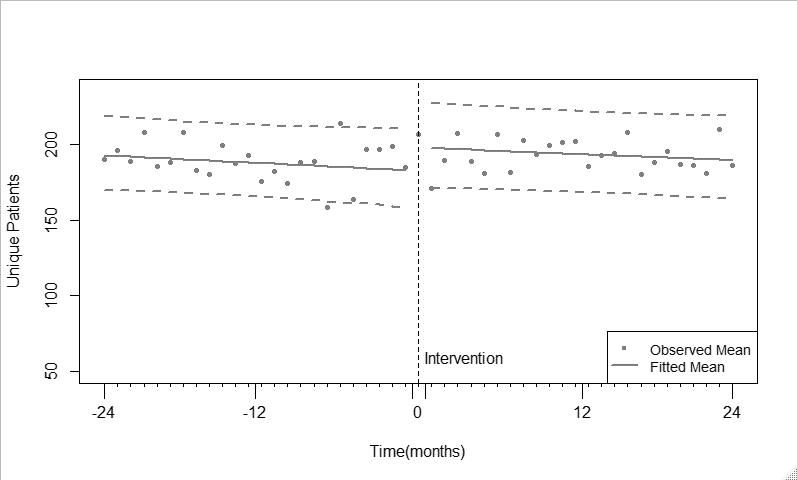 | 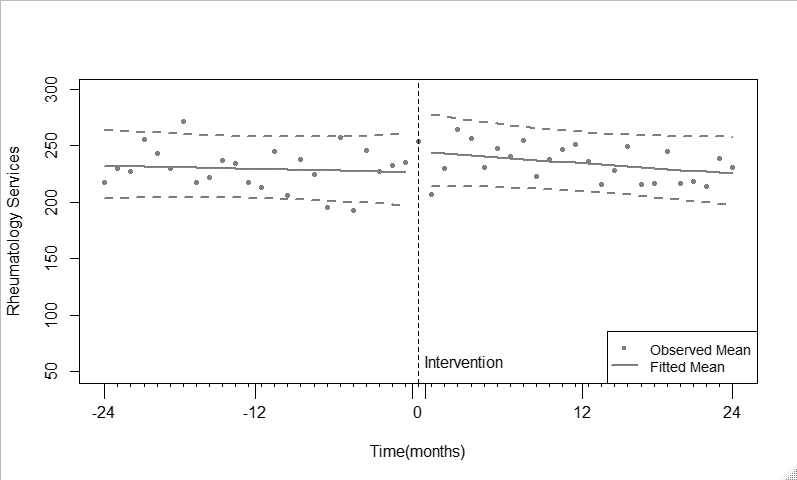 |

**APPENDIX B: Rheumatologist Categorization Algorithm:**

1. Using MSP Billing Data, identify population of interest: Rheumatologists who use G31060.
2. Ensure sufficient data for balanced time series analysis: 24 months both prior to and after the rheumatologist’s first use of the nurse code. 🡪“Ever-Billed” Rheumatologists
3. From “Ever-Billed”, identify rheumatologists billing G31060 at least once per month in every month after the first use of the nurse code. 🡪”Consistent Rheumatologists-A”
4. Identify potentially consistent rheumatologists who are not billing G31060 *every* month:
   1. From “Ever-Billed”, identify rheumatologists with brief interruptions in billing of G31060 (1-3 months).
   2. For those months of interruption in G31060, check to see if the rheumatologist is billing any *other* rheumatology codes during that period.
   3. If they are still active, then the rheumatologist is considered inconsistent. If they are not, treat as vacation/sabbatical/illness, part of normal practice and does not necessarily indicate inconsistency. 🡪”Consistent Rheumatologists-B”
5. Combine “Consistent Rheumatologists-A” and “Consistent Rheumatologists-B” 🡪 “Consistent Rheumatologists”
6. Identify rheumatologists using G31060 with “High Intensity” (>=25 billings/month, on average)
   1. From “Ever-Billed”, compute the *average* number of billings of G31060 per month for each rheumatologist, after their first use of the code.
   2. Filter the rheumatologists to those billing G31060 *at least* 25 times per month, on average 🡪 “High Intensity”
7. Identify rheumatologists who never use the nurse code 🡪 “Status Quo Rheumatologists”
8. Returning to rheumatologists who use the nurse code, identify those that do not have 24 months both prior to and after the rheumatologists first use of the nurse code 🡪 “Insufficient time-series data”

“Consistent” and “High-Intensity” are subsets of “Ever-Billed”.

“Consistent” and “High-Intensity” rheumatologists mostly intersect but “High-Intensity” is not a subset of “Consistent”. A “High-Intensity” rheumatologist could fail the condition for consistency (4b) and still maintain a sufficiently high level of billing that they still meet the intensity condition (6b), as it is an average.

“Status Quo”, “Ever-Billed”, and “Insufficient time-series data” are mutually exclusive categories.
